# Supplementary material for: An international multi-centre study to develop and validate federated learning-based prognostic models for anal cancer
Source: Nat Commun. 2026 Mar 14;17:3956. doi: 10.1038/s41467-026-70297-3 (PMC13133161; doi:10.1038/s41467-026-70297-3)
Supplement: Supplementary file 1 — Supplementary Information [file 41467_2026_70297_MOESM1_ESM.pdf]

# Supplementary Material

## Supplementary Note 1

## TRIPOD checklist for prediction model development

| Section/Topic | | Checklist Item | Page |
|---|---|---|---|
| Title and abstract | | | |
| Title | 1 | Identify the study as developing and/or validating a multivariable prediction model, the target population, and the outcome to be predicted. | 1 |
| Abstract | 2 | Provide a summary of objectives, study design, setting, participants, sample size, predictors, outcome, statistical analysis, results, and conclusions. | 2 |
| Introduction | | | |
| Background and objectives | 3a | Explain the medical context (including whether diagnostic or prognostic) and rationale for developing or validating the multivariable prediction model, including references to existing models. | 3 |
| | 3b | Specify the objectives, including whether the study describes the development or validation of the model or both. | 3-4 |
| Methods | | | |
| Source of data | 4a | Describe the study design or source of data (e.g., randomized trial, cohort, or registry data), separately for the development and validation data sets, if applicable. | 9 |
| | 4b | Specify the key study dates, including start of accrual; end of accrual; and, if applicable, end of follow-up. | 4 |
| Participants | 5a | Specify key elements of the study setting (e.g., primary care, secondary care, general population) including number and location of centres. | 1,9 |
| | 5b | Describe eligibility criteria for participants. | 9 |
| | 5c | Give details of treatments received, if relevant. | 9 |
| Outcome | 6a | Clearly define the outcome that is predicted by the prediction model, including how and when assessed. | 9,10 |
| | 6b | Report any actions to blind assessment of the outcome to be predicted. | N/A |
| Predictors | 7a | Clearly define all predictors used in developing or validating the multivariable prediction model, including how and when they were measured. | 10 |
| | 7b | Report any actions to blind assessment of predictors for the outcome and other predictors. | N/A |
| Sample size | 8 | Explain how the study size was arrived at. | 10,11 |
| Missing data | 9 | Describe how missing data were handled (e.g., complete-case analysis, single imputation, multiple imputation) with details of any imputation method. | 10 |
| Statistical analysis methods | 10a | Describe how predictors were handled in the analyses. | 11 |
| | 10b | Specify type of model, all model-building procedures (including any predictor selection), and method for internal validation. | 11 |
| | 10d | Specify all measures used to assess model performance and, if relevant, to compare multiple models. | 11,12 |
| Risk groups | 11 | Provide details on how risk groups were created, if done. | 12 |
| Results | | | |
| Participants | 13a | Describe the flow of participants through the study, including the number of participants with and without the outcome and, if applicable, a summary of the follow-up time. A diagram may be helpful. | 4 |
| | 13b | Describe the characteristics of the participants (basic demographics, clinical features, available predictors), including the number of participants with missing data for predictors and outcome. | 4 |
| Model development | 14a | Specify the number of participants and outcome events in each analysis. | 4 |
| | 14b | If done, report the unadjusted association between each candidate predictor and outcome. | N/A |
| Model specification | 15a | Present the full prediction model to allow predictions for individuals (i.e., all regression coefficients, and model intercept or baseline survival at a given time point). | 4,5 |
| | 15b | Explain how to the use the prediction model. | 5 |
| Model performance | 16 | Report performance measures (with CIs) for the prediction model. | 5 |
| Discussion | | | |
| Limitations | 18 | Discuss any limitations of the study (such as nonrepresentative sample, few events per predictor, missing data). | 7,8 |
| Interpretation | 19b | Give an overall interpretation of the results, considering objectives, limitations, and results from similar studies, and other relevant evidence. | 6,7 |
| Implications | 20 | Discuss the potential clinical use of the model and implications for future research. | 8 |
| Other information | | | |
| Supplementary information | 21 | Provide information about the availability of supplementary resources, such as study protocol, Web calculator, and data sets. | 9,12,13 |
| Funding | 22 | Give the source of funding and the role of the funders for the present study. | 16 |

## SUPPLEMENTARY NOTE 2

## ATOMCAT CONSORTIUM CENTRES AND ETHICAL APPROVALS

Supplementary Table 1. List of atomCAT consortium participating centres, and corresponding ethical approvals and informed consent status.

| Centre | Approving board / committee | Approval reference number | Approval date | Informed consent status |
|---|---|---|---|---|
| Bank of Cyprus Oncology Centre, Nicosia, Cyprus | Cyprus National Bioethics Committee | ΕΕΒΚ ΕΠ 2021.01.145 | 03/06/2021 | Waived by ethics committee |
| Cambridge University Hospital NHS Foundation Trust, Cambridge, UK | HRA & REC approval for atomCAT2 | 22/WA/0081 | 08/03/2022 | Waived by HRA & REC |
| Champalimaud Foundation, Lisbon, Portugal | Champalimaud Foundation Ethics Committee | 20220118.02 atomCAT2A | 18/01/2022 | Not required by ethics committee |
| Fondazione Policlinico Universitario A.Gemelli IRCCS, Università Cattolica S.Cuore, Rome, Italy | Ethics Committee of the Fondazione Policlinico Universitario Agostino Gemelli IRCCS; Università Cattolica del sacro Cuore | 0027721/21 | 22/07/2021 | Waived by ethics committee |
| Greater Poland Cancer Centre, Poznan, Poland | Poznan University of Medical Sciences Bioethics Committee | KB - 530/21 | 16/06/2021 | Not required by ethics committee |
| Hull University Teaching Hospitals NHS Trust, Hull, UK | HRA & REC approval for atomCAT2 | 22/WA/0081 | 08/03/2022 | Waived by HRA & REC |
| Leeds Teaching Hospitals NHS Trust, Leeds, UK | LeedsCAT Governance Board | LeedsCAT001 | 19/09/2019 | Waived by governance board |
| Maastro Clinic, Maastricht, The Netherlands | Institutional Review Board of MAASTRO Clinic (Dept of Radiotherapy, Faculty of Health Medicine and Lifesciences, Maastricht University Medical Centre+, Netherlands) | P0266 | 25/10/2017 | Waived by institutional review board |
| Oslo University Hospital, Oslo, Norway | Regional Committee for Medical and Health Research Ethics | 2012/2274 | 05/05/2021 | Waived by ethics committee |

| | | | | |
|---|---|---|---|---|
| Oxford University Hospitals NHS Foundation Trust, Oxford, UK | Oxford University Hospital Clinical Audit Team | 6887 | 28/04/2021 | Not required (classified as clinical audit) |
| RWTH Aachen University Medical Centre, Aachen, Germany | Ethics Committee, Faculty of Medicine, RWTH Aachen University, Aachen, Germany | EK 478/21 | 16/12/2021 | Waived by ethics committee |
| The Christie NHS Foundation Trust, Manchester, UK | ukCAT Governance Board | 2022-011 | 04/07/2022 | Waived by governance board |
| The Netherlands Cancer Institute - Antoni van Leeuwenhoek (NKI-AVL), Amsterdam, The Netherlands | NKI-AVL Institutional Review Board | IRBd21-166 | 06/08/2021 | Waived by institutional review board |
| Velindre University NHS Trust, Cardiff, United Kingdom | CardiffCAT Management Committee | 19/WA/0119 | 20/10/2022 | Waived by management committee |
| Liverpool and Macarthur Cancer Therapy Centres, Liverpool, New South Wales, Australia (external validation centre) | New South Wales (NSW) Population & Health Services Research Ethics Committee | 2019/ETH01550 | 30/11/2022 | Waived by ethics committee |
| Goethe University Frankfurt, University Hospital, Frankfurt, Germany (external validation centre) | Ethics Committee University Hospital Frankfurt, Germany | 458/17 | 09/02/2018 | Patient consent obtained as prior general consent for research use |

## Supplementary Note 3

# Additional methodological details

This supplementary note includes additional methodological details regarding the handling of missing data and data standardisation procedures across centres, as per study protocol (Theophanous et al, Diagn Progn Res 2022).

## Data collection and completeness

Relevant patient data were identified and extracted from existing research and clinical databases. Data extraction from databases was carried out in an automated fashion where possible, and additional manual review was implemented where needed. Each participating centre was responsible for ensuring good data quality by spot checking all extracted data to identify any outliers and to make sure the coding system used was correct, according to the data dictionary that was provided by the coordinating team.

Centres aimed for at most 10% missing data for any given data item across their study cohort. If more than 10% of data was missing for an individual data item, imputation techniques were implemented according to the framework set out below (see “Missing Data” section).

Each centre contributed data from a minimum of 40 patients to ensure a representative sample and to achieve a reasonable balance of patient heterogeneity, as well as limit reporting of subgroups with one or only a few patients. See “Data Dictionary” section below for full definition and coding of data items used in the analysis.

## Missing data

For outcome data, complete case analysis was used for each of the three outcomes. That is, if data was missing for a specific outcome for a patient, that patient did not contribute to the corresponding analysis. For potential prognostic factors, a mixed approach was used: if more than 90% of patients per centre had complete data for all factors for a given analysis, then complete case analysis was used as the primary analysis for that centre. If not, missing value imputation was used according to the framework set out below before any models were fitted, and complete case analysis was performed as a robustness check.

Where data for the same data item were systematically missing in two or more centres (>50% data missing for any specific item), the mean from each centre (apart from the centres with missing data) was used to calculate the global “median of means” value for that data item (for continuous data items). This value was assigned to all patients in the centres where the data item is missing. For categorical data items, the frequency of each category across the global cohort for the data item that was missing was calculated (excluding centres with the missing data item). Categories were then assigned to each patient at random in centres where the data item is missing, ensuring the local frequency distribution was the same as the global frequency distribution.

## Data dictionary

Baseline characteristics

- Biological sex [*sex*]: Binary variable
    - 0: Male
    - 1: Female
- Age at the start of radiotherapy (years) [*age*]: Continuous numerical variable
- TNM staging: Categorical variables
    - T stage [*t_stage*]
        - 1: T1
        - 2: T2
        - 3: T3
        - 4: T4

- N stage [*n_stage*]
  - for TNM version 7: 0: N0; 1: N1; 2: N2; 3: N3
  - for TNM version 8: 0: N0; 1: N1a; 2: N1b; 3: N1c
- M stage [*m_stage*]
  - 0: M0
  - 1: M1

- TNM staging version [*tnm_version*]: Discrete numerical variable
- Primary tumour GTV ($cm^3$) [*pr_tumour_gtv*]: Continuous numerical variable
- Histology [*histology*]: Binary variable
  - 0: SCC
  - 1: Basaloid SCC

Treatment-related factors

- Radiotherapy technique [*rt_technique*]: Categorical variable
  - 1: 3D-CRT
  - 2: IMRT
  - 3: VMAT
- Total prescribed dose (in $EQD2_{\alpha/\beta=10Gy}$): Continuous numerical variable
  - To primary tumour [*prescr_dose_prtumour*]
  - To involved lymph nodes [*prescr_dose_invnodes1, prescr_dose_invnodes2*]
  - To elective nodes [*prescr_dose_elenodes1, prescr_dose_elenodes2*]
- Concurrent chemotherapy? [*conc_chemo*]: Binary variable
  - 0: No
  - 1: Yes
- Concurrent chemotherapy–drugs used [*conc_chemo_drugs*]: Categorical variable
  - 0: No chemotherapy
  - 1: Mitomycin C and 5-Fluorouracil
  - 2: Mitomycin C and Capecitabine
  - 3: Cisplatin and 5-Fluorouracil
  - 4: Cisplatin and Capecitabine
  - 5: Other

Outcomes

- Overall survival status [*os_status*]: Binary variable
  - 0: Alive
  - 1: Dead
- Overall survival - follow-up time (days) [*os_fup*]: Discrete numerical variable
  - Calculated in number of days from the first fraction of radiotherapy to either event or censoring, whichever happens first.
- Locoregional failure [*lrf_status*]: Binary variable
  - 0: No
  - 1: Yes
- Site of locoregional failure [*lrf_site*]: Categorical variable
  - 0: No locoregional failure
  - 1: Primary tumour
  - 2: Pelvic lymph nodes/lymph nodes in the primary treatment volume
  - 3: Primary tumour and lymph nodes simultaneous
  - 4: Other
- Locoregional failure - follow-up time (days) [*lrf_fup*]: Discrete numerical variable
  - Calculated in number of days from the first fraction of radiotherapy to either event or censoring, whichever happens first.
- Distant metastasis [*dm_status*]: Binary variable
  - 0: No
  - 1: Yes
- Distant metastasis - follow-up time (days) [*dm_fup*]: Discrete numerical variable
  - Calculated in number of days from the first fraction of radiotherapy to either event or censoring, whichever happens first.

## Supplementary Note 4

### Federated learning architecture

The Vantage6 v2.3.4 software was used to establish the three elements required to carry out an analysis via federated learning. The first component is a “node”, where individual-level patient data is accessed, and local model coefficients are computed. The second component is a trusted coordinating “server”, which handles the communication with the nodes and performs the aggregation of coefficients from all nodes. The final component is a “researcher”, which provides a pre-specified model for training and validation.

At each participating centre, the node was set up on either a physical or a virtual personal computer running either Windows, MacOS or Ubuntu, with an installation of Python (v3.7 or v3.8), Docker Desktop (personal edition), and the Vantage6 v2.3.4 Python library. The source code for the infrastructure implementation is openly accessible [https://github.com/vantage6/vantage6 - Version 2.3.4]. Network connectivity was fully compliant with local institutional policies, and only one secured network port through the institution firewall was enabled for Vantage6 traffic.

The federated Cox algorithm developed by Lu et al.[31] was adapted to the Vantage6 v2.3.4 infrastructure as R scripts (v.3.6.2) and is publicly available on GitHub [https://github.com/IKNL/vtg.coxph]. During federated model training, each participating centre independently fitted a local Cox model on its own data and transmitted only aggregated summary statistics - specifically, the first- and second-order partial derivatives of the log-likelihood (gradient and Hessian) - to the central coordinating server. No individual-level patient data were exchanged. The central aggregator then updated the global parameter vector by summing site-level contributions to the global gradient and Hessian, following the framework described by Lu et al. Model parameters were iteratively updated until convergence, typically achieved within 6-10 global aggregation rounds. Convergence was defined as a change in the global log-likelihood of less than $1\times10^{-5}$ between consecutive iterations. This iterative optimisation ensured that the federated solution was mathematically equivalent to the pooled maximum-likelihood estimator under the Breslow approximation, while maintaining full data privacy.

The validation algorithm used is publicly available on GitHub (https://github.com/MaastrichtU-CDS/vtg.coxph_val).

Medical Data Works BV (MDW, https://medicaldataworks.nl/) provided and maintained the DL infrastructure that was used to conduct the atomCAT2 DL analysis.

Scripts for model coefficient computation and leave-one-centre-out model validation were packaged as application containers via Docker and were locally executed at each centre. Additional R scripts for extraction of summary statistics as well as model discrimination were used for both the primary and validation cohorts (manually executed by local researchers).

## Supplementary Note 5

## Supplementary Tables and Figures

Supplementary Table 2. Summary statistics for patient and treatment characteristics for the primary cohort. SD: standard deviation; GTV: Gross tumour volume; SCC: Squamous cell carcinoma; 3D-CRT: 3D conformal radiotherapy; IMRT: Intensity modulated radiotherapy; VMAT: Volumetric modulated arc therapy; MMC: Mitomycin C; 5FU: 5-fluorouracil; Cap: Capecitabine; Cispl: Cisplatin. *Centre 10 primary tumour GTV imputed for 53 patients, and Centre 12 primary tumour GTV imputed for 37 patients.

| | Centre 1 | Centre 2 | Centre 3 | Centre 4 | Centre 5 | Centre 6 | Centre 7 | Centre 8 | Centre 9 | Centre 10 | Centre 11 | Centre 12 | Centre 13 | Centre 14 | Overall cohort |
|---|---|---|---|---|---|---|---|---|---|---|---|---|---|---|---|
| **Number of patients** | **274** | **210** | **150** | **128** | **112** | **107** | **82** | **77** | **61** | **53** | **50** | **48** | **44** | **32** | **1428** |
| **Treatment period** | 2011-2022 | 2015-2021 | 2014-2022 | 2013-2017 | 2016-2021 | 2013-2017 | 2009-2021 | 2008-2017 | 2008-2021 | 2004-2017 | 2010-2022 | 2016-2018 | 2013-2022 | 2017-2019 | 2004-2022 |
| **Sex** | | | | | | | | | | | | | | | |
| **Male** | 72 (26%) | 63 (30%) | 48 (32%) | 35 (27%) | 27 (24%) | 26 (24%) | 35 (43%) | 33 (43%) | 21 (34%) | 23 (43%) | 13 (26%) | 19 (40%) | 9 (20%) | 11 | 435 (30%) |
| **Female** | 202 (74%) | 147 (70%) | 102 (68%) | 93 (73%) | 85 (76%) | 81 (76%) | 47 (57%) | 44 (57%) | 40 (66%) | 30 (57%) | 37 (74%) | 29 (60%) | 35 (80%) | 21 | 993(70%) |
| **Age at the start of radiotherapy (years)** | | | | | | | | | | | | | | | |
| **Mean** | 63.1 | 61.6 | 63.3 | 62.8 | 62.8 | 62.0 | 59.5 | 61.3 | 62.0 | 63.6 | 63.9 | 62.1 | 64.8 | 58.6 | 62.4 |
| **(sd, range)** | (11.3, 35-94) | (10.9, 29-87) | (12.3, 29-90) | (10.6, 40-89) | (11.0, 34-86) | (11.4, 31-85 | (12.2, 35-86) | (10.4, 29-85) | (9.7, 44-83) | (10.5, 39-84) | (10.3, 42-84) | (9.5, 38-78) | (12.6, 39-90) | (17.6, 0-83) | (11.3, 29-94) |
| **Age <50** | 35 (13%) | 30 (14%) | 20 (13%) | 25 (20%) | 13 (11%) | 19 (18%) | 15 (18%) | 6 (8%) | 8 (13%) | 5 (9%) | 3 (6%) | 5 (10%) | 6 (13%) | 7 (22%) | 197 (14%) |
| **Age 50-69** | 156 (57%) | 126 (60%) | 80 (54%) | 74 (58%) | 69 (62%) | 58 (54%) | 51 (62%) | 54 (70%) | 39 (64%) | 32 (61%) | 29 (58%) | 31 (65%) | 21 (48%) | 15 (47%) | 835 (58%) |
| **Age >= 70** | 83 (30%) | 54 (26%) | 50 (33%) | 29 (22%) | 30 (27%) | 30 (28%) | 16 (20%) | 17 (22%) | 14 (23%) | 16 (30%) | 18 (36%) | 12 (25%) | 17 (39%) | 10 (31%) | 396 (28%) |
| **T stage at diagnosis** | | | | | | | | | | | | | | | |
| **T1** | 33 (12% | 19 (9%) | 20 (13%) | 15 (12%) | 15 (13%) | 16 (15%) | 15 (18%) | 12 (16%) | 5 (8%) | 2 (4%) | 8 (16%) | 2 (4%) | 5 (11%) | 2 (6%) | 169 (12%) |
| **T2** | 120 (44%) | 95 (45%) | 78 (52%) | 58 (45%) | 45 (40%) | 52 (49%) | 38 (46%) | 44 (57%) | 18 (30%) | 19 (36%) | 12 (24%) | 32 (67%) | 21 (48%) | 13 (41%) | 645 (45%) |
| **T3** | 55 (20%) | 57 (27%) | 27 (18%) | 24 (19%) | 29 (26%) | 25 (23%) | 20 (24%) | 11 (14%) | 32 (52%) | 16 (30%) | 12 (24%) | 10 (21%) | 13 (30%) | 8 (25%) | 339 (24%) |
| **T4** | 66 (24%) | 39 (19%) | 25 (17%) | 31 (24%) | 23 (21%) | 14 (13%) | 9 (11%) | 10 (13%) | 6 (10%) | 16 (30%) | 18 (36%) | 4 (8%) | 5 (11%) | 9 (28%) | 275 (19%) |
| **N stage at diagnosis** | | | | | | | | | | | | | | | |
| **N0** | 148 (54%) | 96 (46%) | 79 (53%) | 69 (54%) | 45 (40%) | 53 (50%) | 41 (50%) | 33 (43%) | 36 (59%) | 21 (40%) | 15 (30%) | 28 (58%) | 19 (43%) | 14 | 697 (49%) |
| **N+** | 126 (46%) | 114 (54%) | 71 (47%) | 59 (46%) | 67 (60%) | 54 (50%) | 41 (50%) | 44 (57%) | 25 (41%) | 32 (60%) | 35 (70%) | 20 (42%) | 25 (57%) | 18 | 731 (51%) |
| **M stage at diagnosis** | | | | | | | | | | | | | | | |
| **M0** | 271 (99%) | 191 (91%) | 147 (98%) | 126 (98%) | 112 (100%) | 106 (99%) | 82 (100%) | 77 (100%) | 61 (100%) | 53 (100%) | 48 (96%) | 48 (100%) | 37 (84%) | 31 (97%) | 1390 (97%) |
| **M1** | 3 (1%) | 19 (9%) | 3 (2%) | 2 (2%) | 0 (0%) | 1 (1%) | 0 (0%) | 0 (0%) | 0 (0%) | 0 (0%) | 2 (4%) | 0 (0%) | 7 (16%) | 1 (3%) | 38 (3%) |
| **Primary tumour GTV (cm3)** | | | | | | | | | | | | | | | |
| **Mean** | 120.5 | 53.9 | 68.6 | 76.1 | 64.4 | 61.1 | 23.9 | 59.2 | 73.6 | 62.0 | 50.3 | 56.5 | 43.6 | 39.2 | 70.6 |
| **(sd, range)** | (101.9, 2.9-651.2) | (86.0, 1.1-974.4) | (68.6, 1.79-446.0) | (67.5, 4.1-459.4) | (68.7, 0.3-314.6) | (93.7, 0.7-633.2) | (32.2, 1.1-212.0) | (73.8, 0.8-433.0) | (67.9, 1.8-328.3) | (0, 62.0-62.0) | (34.1, 8.8-143.8) | (14.7, 10.0-85.0) | (69.60, 1.9-357.4) | (41.3, 1.2-162.9) | (157.3, 0.6-974.4) |
| **Median** | 51.7 | 30.6 | 45.5 | 57.8 | 37.6 | 25.4 | 12.6 | 30.3 | 45.7 | 62.0 | 36.4 | 62.0 | 19.8 | 23.1 | 41.5 |
| **(Q1, Q3)** | (26.3, 102.3) | (13.0, 60.6) | (27.1, 83.1) | (37.0, 81.9) | (17.1, 85.7) | (9.1, 67.0) | (4.2, 30.9) | (15.5, 67.0) | (28.0, 96.7) | (62.0, 62.0) | (26.0, 66.1) | (62.0, 62.0) | (8.1, 42.1) | (11.3, 57.1) | |
| **GTV delineation** | Primary tumour only for the majority. For some patients, primary tumour and anal canal at the level of the tumour. | Primary tumour only | Primary tumour only | Primary tumour and anal canal at the level of the tumour | Primary tumour only | Primary tumour only | Primary tumour and anal canal at the level of the tumour | Primary tumour only | Primary tumour only | N/A - Used consortium mean GTV | Primary tumour only | Used consortium mean GTV for most patients. Where GTV is available: Primary tumour only. | Primary tumour only | Primary tumour only | |
| **Histology** | | | | | | | | | | | | | | | |
| **SCC** | 221 (81%) | 193 (92%) | 132 (88%) | 107 (84%) | 95 (85%) | 87 (81%( | 80 (98%) | 76 (99%) | 57 (93%) | 37 (70%) | 41 (82%) | 42 (88%) | 33 (75%) | 32 | 1233 (86%) |
| **Basaloid SCC** | 53 (19%) | 17 (8%) | 18 (12%) | 21 (16%) | 17 (15%) | 20 (19%) | 2 (2%) | 1 (1%) | 4 (7%) | 16 (30%) | 9 (18%) | 6 (12%) | 11 (25%) | 0 | 195 (14%) |
| **Primary tumour dose (EQD2 α/β=10)** | | | | | | | | | | | | | | | |
| **Mean** | 50.6 | 52.5 | 51.8 | 56.4 | 51.4 | 51.2 | 55.3 | 60.1 | 53.5 | 52.5 | 54.0 | 59.6 | 56.9 | 50.9 | 53.2 |
| **(sd, range)** | (4.1, 19.5-62.0) | (2.5, 49.6-65.2) | (3.8, 40.7-63.6) | (2.0, 54.0-58.1) | (4.5, 40.7-62.6) | (1.6, 49.6-52.8) | (2.0, 49.6-60.0) | (2.5, 54.0-66.0) | (3.4, 49.6-60.0) | (2.1, 54.6-58.4) | (3.0, 49.6-60.0) | (2.3, 58.4-63.7) | (4.2, 44.3-62.0) | (2.8, 40.6-54.3) | (4.2, 40.7-66.0) |
| **Radiotherapy technique** | | | | | | | | | | | | | | | |
| **3D-CRT** | 0 (0%) | 0 (0%) | 3 (2%) | 42 (33%) | 0 (0%) | 0 (0%) | 0 (0%) | 24 (31%) | 20 (33%) | 25 (47%) | 3 (6%) | 0 (0%) | 0 (0%) | 0 (0%) | 117 (8%) |
| **IMRT / VMAT** | 274 (100%) | 210 (100%) | 147 (98%) | 86 (67%) | 112 (100%) | 107 (100%) | 82 (100%) | 53 (69%) | 41 (67%) | 28 (53%) | 47 (94%) | 48 (100%) | 44 (100%) | 32 (100%) | 1311 (92%) |
| **Chemotherapy regimen** | | | | | | | | | | | | | | | |
| **No chemotherapy** | 7 (3%) | 1 (<1%) | 13 (9%) | 9 (7%) | 20 (18%) | 1 (<1%) | 6 (7%) | 12 (16%) | 6 (10%) | 0 (0%) | 0 (0%) | 1 (2%) | 6 (14%) | 1 | 83 (6%) |
| **MMC and 5FU** | 168 (61%) | 176 (84%) | 100 (67%) | 114 (88%) | 77 (69%) | 21 (20%) | 67 (82%) | 0 (0%) | 49 (80%) | 48 (90%) | 44 (88%) | 0 (0%) | 32 (73%) | 31 | 927 (65%) |
| **MMC and Cap** | 80 (29%) | 27 (13%) | 33 (22%) | 1 (<1%) | 14 (13%) | 84 (79%) | 7 (9%) | 64 (83%) | 0 (0%) | 3 (6%) | 0 (0%) | 47 (98%) | 5 (11%) | 0 (0%) | 365 (26%) |
| **Cispl and 5FU** | 0 (0%) | 1 (<1%) | 0 (0%) | 4 (3%) | 0 (0%) | 0 (0%) | 0 (0%) | 0 (0%) | 5 (8%) | 0 (0%) | 2 (4%) | 0 (0%) | 0 (0%) | 0 (0%) | 12 (<1%) |
| **Cispl and Cap** | 0 (0%) | 0 (0%) | 1 (<1%) | 0 (0%) | 1 (<1%) | 0 (0%) | 0 (0%) | 0 (0%) | 0 (0%) | 0 (0%) | 0 (0%) | 0 (0%) | 1 (2%) | 0 (0%) | 3 (<1%) |
| **Other** | 19 (7%) | 5 (2%) | 3 (2%) | 0 (0%) | 0 (0%) | 1 (<1%) | 2 (2%) | 1 (1%) | 1 (2%) | 2 (4%) | 4 (8%) | 0 (0%) | 0 (0%) | 0 (0%) | 38 (3%) |

Supplementary Table 3. Summary statistics for patient and treatment characteristics, and summary of survival statistics from the two validation cohorts. SD: standard deviation; GTV: Gross tumour volume; SCC: Squamous cell carcinoma; MMC: Mitomycin C; 5FU: 5-fluorouracil; Cap: Capecitabine; Cispl: Cisplatin. *Validation centre 2 primary tumour GTV imputed for 21 patients.

| | | Validation Centre 1 | Validation Centre 2 | Overall validation cohort |
|---|---|---|---|---|
| **Number of patients** | | **174** | **103** | **277** |
| **Treatment period** | | 2008-2020 | 2010-2023 | 2008-2023 |
| **Sex** | | | | |
| | **Male** | 82 (47%) | 32 (31%) | 114 (41%) |
| | **Female** | 92 (53%) | 71 (69%) | 163 (59%) |
| **Age at the start of radiotherapy (years)** | | | | |
| | **Mean** | 59.5 | 63.4 | 61.0 |
| | **(sd, range)** | (12.1, 26-87) | (12.9, 21-92) | 12.5 (21-92) |
| **T stage at diagnosis** | | | | |
| | **T1** | 44 (25%) | 11 (11%) | 55 (20%) |
| | **T2** | 79 (45%) | 53 (51%) | 132 (48%) |
| | **T3** | 40 (23%) | 26 (25%) | 66 (24%) |
| | **T4** | 11 (6%) | 13 (13%) | 24 (9%) |
| **N stage at diagnosis** | | | | |
| | **N0** | 101 (58%) | 54 (52%) | 155 (56%) |
| | **N+** | 73 (42%) | 49 (48%) | 122 (44%) |
| **M stage at diagnosis** | | | | |
| | **M0** | 173 (99%) | 99 (96%) | 272 (98%) |
| | **M1** | 1 (<1%) | 4 (4%) | 5 (2%) |
| **Primary tumour GTV (cm3)** | | | | |
| | **Mean** | 26.4 | 62.2 | 39.7 |
| | **(sd, range)** | (35.3, 1.4-314.6) | (53.8, 2.31-263.59) | (46.4, 1.4-314.6) |
| | **Median** | 15.4 | 53.5 | 29.6 |
| | **(Q1, Q3)** | (6.6, 34.6) | (27.5, 63.9) | |
| **GTV delineation** | | | | |
| | | Primary tumour only | Primary tumour only for majority. For some patients, primary tumour and anal canal at the level of the tumour. For small number of patients with missing GTV, mean GTV of cohort was used. | |
| **Histology** | | | | |
| | **SCC** | 151 (87%) | 99 (96%) | 250 (90%) |
| | **Basaloid SCC** | 23 (13%) | 4 (4%) | 27 (10%) |
| **Primary tumour dose (EQD2 α/β=10)** | | | | |
| | **Mean** | 56.4 | 53.4 | 55.3 |
| | **(sd, range)** | (3.1, 33.6-63.7) | (2.1, 49.6-62.0) | (3.1, 33.6-63.7) |
| **Radiotherapy technique** | | | | |
| | **3D-CRT** | 32 (18%) | 6 (6%) | 38 (14%) |
| | **IMRT / VMAT** | 142 (82%) | 97 (94%) | 239 (86%) |
| **Chemotherapy regimen** | | | | |
| | **No chemotherapy** | 5 (3%) | 47 (46%) | 52 (19%) |
| | **MMC and 5FU** | 160 (92%) | 34 (33%) | 194 (70%) |
| | **MMC and Cap** | 1 (<1%) | 21 (20%) | 22 (8%) |
| | **Cispl and 5FU** | 2 (1%) | 1 (1%) | 3 (1%) |
| | **Cispl and Cap** | 0 (0%) | 0 (0%) | 0 (0%) |
| | **Other** | 6 (3%) | 0 (0%) | 6 (2%) |
| **Number of events** | | | | |
| | **Deaths** | 29 (17%) | 15 (15%) | 44 (16%) |
| | **Locoregional failures** | 22 (13%) | 10 (10%) | 32 (12%) |
| | **Distant metastases** | 19 (11%) | 11 (11%) | 40 (14%) |
| **Estimated overall survival rates** | | | | |
| | **2-year OS** | 89% | 89% | 89% |
| | **3-year OS** | 87% | 87% | 87% |
| | **5-year OS** | 80% | 76% | 79% |
| **Estimated locoregional control rates** | | | | |
| | **2-year LRC** | 91% | 89% | 90% |
| | **3-year LRC** | 91% | 87% | 90% |
| | **5-year LRC** | 82% | 87% | 84% |
| **Estimated freedom from distant metastases rates** | | | | |
| | **2-year FFDM** | 89% | 90% | 89% |
| | **3-year FFDM** | 89% | 90% | 89% |
| | **5-year FFDM** | 87% | 90% | 88% |

Supplementary Table 4. Survival statistics, stratified by outcome and centre. OS: overall survival; LRC: locoregional control; FFDM: freedom from distant metastases; N/A: No 5-year follow-up information available.

| | Centre 1 | Centre 2 | Centre 3 | Centre 4 | Centre 5 | Centre 6 | Centre 7 | Centre 8 | Centre 9 | Centre 10 | Centre 11 | Centre 12 | Centre 13 | Centre 14 | Overall cohort |
|---|---|---|---|---|---|---|---|---|---|---|---|---|---|---|---|
| Number of patients | 274 | 210 | 150 | 128 | 112 | 107 | 82 | 77 | 61 | 53 | 50 | 48 | 44 | 32 | 1428 |
| Number of events | | | | | | | | | | | | | | | |
| Deaths | 54 (20%) | 45 (21%) | 35 (23%) | 20 (16%) | 13 (12%) | 18 (17%) | 14 (17%) | 21 (27%) | 17 (28%) | 22 (42%) | 5 (10%) | 11 (23%) | 6 (14%) | 5 (16%) | 286 (20%) |
| Locoregional failures | 43 (16%) | 34 (16%) | 25 (17%) | 13 (10%) | 15 (13%) | 14 (13%) | 13 (16%) | 13 (17%) | 10 (16%) | 10 (19%) | 8 (16%) | 12 (25%) | 4 (9%) | 2 (6%) | 214 (15%) |
| Distant metastases | 40 (15%) | 25 (12%) | 17 (11%) | 7 (5%) | 5 (4%) | 9 (8%) | 12 (15%) | 10 (13%) | 5 (8%) | 9 (17%) | 5 (10%) | 7 (15%) | 7 (16%) | 5 (16%) | 163 (11%) |
| Estimated overall survival rates | | | | | | | | | | | | | | | |
| 2-year OS | 86% | 87% | 82% | 93% | 91% | 90% | 90% | 83% | 87% | 83% | 97% | 83% | 90% | 94% | 88% |
| 3-year OS | 81% | 83% | 78% | 92% | 88% | 83% | 82% | 75% | 80% | 75% | 97% | 78% | 86% | 80% | 83% |
| 5-year OS | 74% | 76% | 69% | 87% | 85% | N/A | 82% | 71% | 67% | 66% | 81% | 69% | 82% | N/A | 76% |
| Estimated locoregional control rates | | | | | | | | | | | | | | | |
| 2-year LRC | 84% | 84% | 83% | 91% | 88% | 88% | 83% | 89% | 86% | 83% | 80% | 78% | 89% | 100% | 86% |
| 3-year LRC | 82% | 82% | 80% | 90% | 85% | 85% | 81% | 89% | 83% | 83% | 80% | 73% | 89% | 75% | 83% |
| 5-year LRC | 81% | 81% | 78% | 89% | 85% | N/A | 81% | 75% | 76% | 80% | 80% | 73% | 89% | N/A | 81% |
| Estimated freedom from distant metastases rates | | | | | | | | | | | | | | | |
| 2-year FFDM | 87% | 88% | 87% | 96% | 96% | 92% | 87% | 86% | 91% | 84% | 87% | 86% | 90% | 91% | 89% |
| 3-year FFDM | 84% | 87% | 86% | 95% | 96% | 90% | 85% | 86% | 91% | 84% | 87% | 84% | 86% | 57% | 87% |
| 5-year FFDM | 82% | 84% | 84% | 94% | 94% | N/A | 85% | 86% | 91% | 84% | 87% | 84% | 80% | N/A | 86% |

Supplementary Figure 1. Observed weighted mean (a) overall survival, (b) locoregional control and (c) freedom from distant metastases rates at two, three, and five years, stratified into predicted high-risk and low-risk groups, across all 14 participating centres. Shaded areas represent the weighted mean 95% confidence intervals. Source data are provided as a Source Data file.

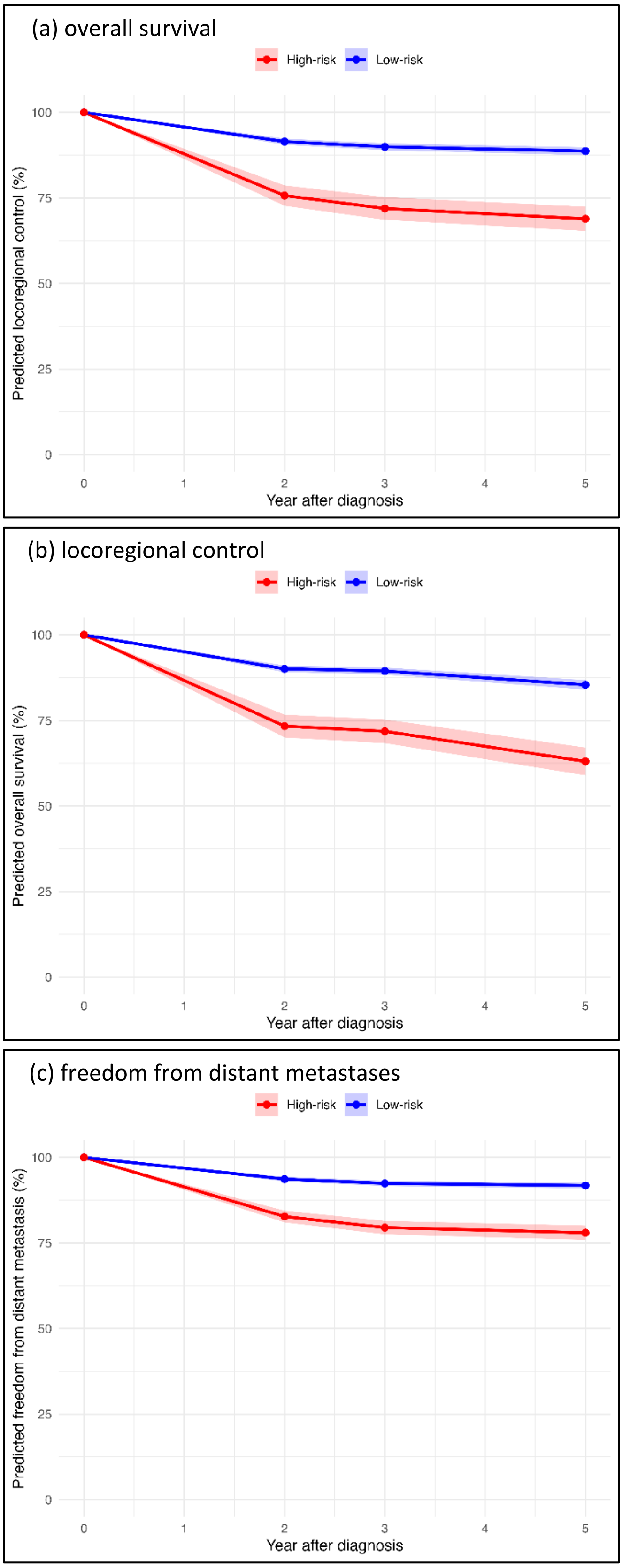

Supplementary Table 5. Reference outcome rates required for individual patient risk prediction using the federated multivariable outcome models that have been developed (see Table 3). To calculate these, all categorical factors were set to 0, age at the start of radiotherapy was set to 35 years, prescribed dose to the primary tumour was set to 40 $EQD2_{\alpha/\beta=10Gy}$, and $log_{10}$ of GTV was set to 0.02572.

| Reference outcome rates | Overall survival | Locoregional control | Freedom from distant metastases |
|---|---|---|---|
| 2 years | 0.916 | 0.976 | 0.973 |
| 3 years | 0.911 | 0.971 | 0.968 |
| 5 years | 0.875 | 0.967 | 0.965 |

Supplementary Table 6. Extended summary of results from the global validation and the leave-one-centre-out validation of the overall survival, locoregional control, and freedom from distant metastases models, in the primary cohort. For the leave-one-centre-out validation, each model was trained on all but one cohort, and subsequently validated on the last, independent cohort.

| Centre | Number of patients | Overall survival | | Locoregional control | | Freedom from distant metastases | |
|---|---|---|---|---|---|---|---|
| | | Global model c-index | Leave-one-centre-out validation c-index | Global model c-index | Leave-one-centre-out validation c-index | Global model c-index | Leave-one-centre-out validation c-index |
| 1 | 274 | 0.64 | 0.64 | 0.68 | 0.67 | 0.66 | 0.64 |
| 2 | 210 | 0.67 | 0.66 | 0.74 | 0.74 | 0.71 | 0.66 |
| 3 | 150 | 0.74 | 0.71 | 0.75 | 0.69 | 0.73 | 0.71 |
| 4 | 128 | 0.71 | 0.70 | 0.73 | 0.73 | 0.70 | 0.69 |
| 5 | 112 | 0.62 | 0.59 | 0.71 | 0.70 | 0.82 | 0.79 |
| 6 | 107 | 0.73 | 0.73 | 0.76 | 0.75 | 0.73 | 0.73 |
| 7 | 82 | 0.71 | 0.70 | 0.68 | 0.68 | 0.65 | 0.65 |
| 8 | 77 | 0.68 | 0.68 | 0.62 | 0.60 | 0.73 | 0.73 |
| 9 | 61 | 0.81 | 0.81 | 0.73 | 0.69 | 0.82 | 0.80 |
| 10 | 53 | 0.59 | 0.57 | 0.66 | 0.65 | 0.53 | 0.44 |
| 11 | 50 | 0.61 | 0.61 | 0.59 | 0.57 | 0.37 | 0.33 |
| 12 | 48 | 0.63 | 0.59 | 0.67 | 0.63 | 0.61 | 0.59 |
| 13 | 44 | 0.76 | 0.73 | 0.78 | 0.77 | 0.65 | 0.53 |
| 14 | 32 | 0.66 | 0.64 | 0.79 | 0.79 | 0.50 | 0.50 |
| Weighted mean | | 0.68 | 0.67 | 0.71 | 0.69 | 0.69 | 0.66 |

Supplementary Table 7. Summary of results from the secondary overall survival models. CI: Confidence interval; GTV: Gross tumour volume; Gy: Gray; SCC: Squamous cell carcinoma; IMRT: Intensity modulated radiotherapy; VMAT: Volumetric modulated arc therapy; 3D-CRT: 3D conformal radiotherapy.

| | Primary model | Secondary model 1 (Staging risk) | Secondary model 2 (Age categorical) | Secondary model 3 (Age squared) | Secondary model 4 (GTV categorical) | Secondary model 5 (GTV complete case) | Secondary model 6 (Performance status) | Secondary model 7 (Completed treatment) | Secondary model 8 (Overall treatment time) | Secondary model 9 (No GTV) | Secondary model 10 (No T stage) |
|---|---|---|---|---|---|---|---|---|---|---|---|
| **Weighted mean c-index** | 0.68 | 0.68 | 0.69 | 0.68 | 0.68 | 0.69 | 0.70 | 0.70 | 0.69 | 0.67 | 0.68 |
| **Factor** | **Hazard ratio (95% CI)** | | | | | | | | | | |
| Nodal involvement (N+ relative to N0) | 1.45 (1.11-1.89) | - | 1.45 (1.11-1.88) | 1.45 (1.12-1.89) | 1.42 (1.09-1.85) | 1.46 (1.11-1.92) | 1.56 (1.14-2.12) | 1.50 (1.07-2.11) | 1.48 (1.10-1.97) | 1.54 (1.19-2.00) | 1.56 (1.20-2.02) |
| T stage (T3-4 relative to T1-2) | 1.42 (1.07-1.89) | - | 1.39 (1.05-1.85) | 1.41 (1.06-1.88) | 1.41 (1.06-1.89) | 1.44 (1.07-1.95) | 1.28 (0.92-1.79) | 1.47 (1.02-2.10) | 1.49 (1.09-2.05) | 1.92 (1.49-2.48) | - |
| Staging risk (High risk - [T4 Nany] or [Tany N+] relative to Low risk - [T1 or T2 or T3] and N0) | - | 1.49 (1.14-1.96) | - | - | - | - | - | - | - | - | - |
| Sex (Female relative to male) | 0.65 (0.51-0.83) | 0.64 (0.50-0.82) | 0.65 (0.51-0.82) | 0.65 (0.51-0.83) | 0.66 (0.52-0.85) | 0.66 (0.52-0.85) | 0.69 (0.52-0.91) | 0.56 (0.42-0.76) | 0.67 (0.52-0.88) | 0.65 (0.51-0.82) | 0.64 (0.50-0.82) |
| Age at the start of radiotherapy | | | | | | | | | | | |
| Linear (per 10 years) | 1.20 (1.07-1.34) | 1.21 (1.08-1.35) | - | - | 1.22 (1.09-1.36) | 1.19 (1.06-1.33) | 1.22 (1.07-1.40) | 1.25 (1.09-1.45) | 1.22 (1.08-1.38) | 1.21 (1.08-1.35) | 1.21 (1.08-1.35) |
| Categorical (50-69 relative to <50) | - | - | 1.04 (0.71-1.52) | - | - | - | - | - | - | - | - |
| Categorical (>=70 relative to <50) | - | - | 1.65 (1.11-2.46) | - | - | - | - | - | - | - | - |
| Squared | - | - | - | 1.00 (1.00-1.00) | - | - | - | - | - | - | - |
| Gross tumour volume (cm3) | | | | | | | | | | | |
| Log10 | 2.02 (1.47-2.76) | 2.41 (1.81-3.20) | 2.05 (1.50-2.81) | 2.02 (1.48-2.77) | - | 2.03 (1.47-2.79) | 2.13 (1.48-3.06) | 2.16 (1.47-3.18) | 2.00 (1.43-2.79) | - | 2.41 (1.82-3.19) |
| Categorical (50-99.99 relative to <49.99) | - | - | - | - | 1.59 (1.18-2.16) | - | - | - | - | - | - |
| Categorical (100-149.99 relative to <49.99) | - | - | - | - | 1.23 (0.75-2.00) | - | - | - | - | - | - |
| Categorical (>=150 relative to <49.99) | - | - | - | - | 2.95 (2.02-4.32) | - | - | - | - | - | - |
| Prescribed dose to primary tumour (per 10 Gy) | 0.96 (0.71-1.29) | 1.00 (0.74-1.36) | 0.97 (0.72-1.31) | 0.96 (0.71-1.29) | 0.98 (0.73-1.31) | 0.97 (0.72-1.32) | 0.97 (0.68-1.38) | 0.85 (0.54-1.34) | 0.84 (0.61-1.17) | 0.92 (0.69-1.24) | 1.00 (0.74-1.34) |
| Histology (Basaloid SCC relative to SCC) | 0.88 (0.61-1.28) | 0.88 (0.61-1.28) | 0.89 (0.61-1.28) | 0.89 (0.61-1.29) | 0.89 (0.61-1.29) | 0.82 (0.53-1.25) | 0.22 (0.59-1.43) | 1.02 (0.64-1.63) | 0.85 (0.55-1.30) | 0.87 (0.60-1.26) | 0.88 (0.61-1.28) |
| Chemotherapy regimen (all relative to no chemotherapy) | | | | | | | | | | | |
| Mitomycin-based chemotherapy | 0.35 (0.23-0.53) | 0.36 (0.24-0.55) | 0.32 (0.23-0.52) | 0.36 (0.24-0.55) | 0.38 (0.25-0.57) | 0.34 (0.23-0.52) | 0.40 (0.25-0.65) | 0.36 (0.23-0.58) | 0.37 (0.24-0.58) | 0.40 (0.24-0.60) | 0.36 (0.24-0.55) |
| Cisplatin-based chemotherapy | 0.32 (0.11-0.92) | 0.33 (0.11-0.96) | 0.33 (0.11-0.96) | 0.33 (0.11-0.96) | 0.35 (0.12-1.01) | 0.31 (0.11-0.89) | 0.36 (0.12-1.08) | 0.35 (0.12-1.03) | 0.35 (0.15-1.04) | 0.41 (0.14-1.18) | 0.33 (0.11-0.95) |
| Other chemotherapy regimens | 0.81 (0.42-1.56) | 0.82 (0.42-1.59) | 0.82 (0.42-1.59) | 0.82 (0.42-1.60) | 0.88 (0.45-1.71) | 0.77 (0.38-1.56) | 0.88 (0.43-1.82) | 0.77 (0.29-2.06) | 0.92 (0.44-1.91) | 0.86 (0.44-1.66) | 0.86 (0.45-1.67) |
| Radiotherapy technique (IMRT/VMAT relative to 3D-CRT) | 0.96 (0.67-1.39) | 0.99 (0.68-1.43) | 0.95 (0.66-1.37) | 0.96 (0.67-1.38) | 0.94 (0.65-1.35) | 1.09 (0.70-1.69) | 1.05 (0.63-1.74) | 1.11 (0.66-1.85) | 1.10 (0.67-1.83) | 0.89 (0.62-1.28) | 0.99 (0.69-1.43) |
| Performance status | | | | | | | | | | | |
| 1 relative to 0 | - | - | - | - | - | - | 1.47 (1.07-2.01) | - | - | - | - |
| 2 or 3 or 4 relative to 0 | - | - | - | - | - | - | 1.94 (1.21-3.12) | - | - | - | - |
| Completed treatment (Yes relative to No) | - | - | - | - | - | - | - | 0.72 (0.39-1.31) | - | - | - |
| Overall treatment time (days) | - | - | - | - | - | - | - | - | 0.99 (0.97-1.01) | - | - |

Supplementary Table 8. Summary of results from the secondary locoregional control models. CI: Confidence interval; GTV: Gross tumour volume; Gy: Gray; SCC: Squamous cell carcinoma; IMRT: Intensity modulated radiotherapy; VMAT: Volumetric modulated arc therapy; 3D-CRT: 3D conformal radiotherapy.

| | Primary model | Secondary model 1 (Staging risk) | Secondary model 2 (Age categorical) | Secondary model 3 (Age squared) | Secondary model 4 (GTV categorical) | Secondary model 5 (GTV complete case) | Secondary model 6 (Performance status) | Secondary model 7 (Completed treatment) | Secondary model 8 (Overall treatment time) | Secondary model 9 (No GTV) | Secondary model 10 (No T stage) |
|---|---|---|---|---|---|---|---|---|---|---|---|
| **Weighted mean c-index** | 0.71 | 0.71 | 0.71 | 0.71 | 0.71 | 0.71 | 0.72 | 0.73 | 0.72 | 0.68 | 0.71 |
| **Factor** | **Hazard ratio (95% CI)** | | | | | | | | | | |
| Nodal involvement (N+ relative to N0) | 1.24 (0.92-1.68) | - | 1.25 (0.92-1.69) | 1.24 (0.92-1.68) | 1.22 (0.90-1.65) | 1.25 (0.91-1.70) | 1.33 (0.96-1.84) | 1.33 (0.92-1.92) | 1.27 (0.92-1.75) | 1.37 (1.02-1.84) | 1.33 (0.99-1.79) |
| T stage (T3-4 relative to T1-2) | 1.46 (1.05-2.03) | - | 1.44 (1.03-2.01) | 1.46 (1.04-2.03) | 1.48 (1.06-2.08) | 1.40 (0.99-1.97) | 1.37 (0.95-1.97) | 1.55 (1.04-2.31) | 1.40 (0.98-1.99) | 2.19 (1.63-2.94) | - |
| Staging risk (High risk: [T4 Nany] or [Tany N+] relative to Low risk: [T1 or T2 or T3] and N0) | - | 1.21 (0.88-1.65) | - | - | - | - | - | - | - | - | - |
| Sex (Female relative to male) | 0.56 (0.43-0.73) | 0.55 (0.42-0.73) | 0.56 (0.43-0.74) | 0.56 (0.43-0.73) | 0.57 (0.43-0.74) | 0.56 (0.42-0.73) | 0.59 (0.44-0.79) | 0.52 (0.38-0.73) | 0.60 (0.45-0.80) | 0.55 (0.42-0.72) | 0.55 (0.42-0.72) |
| Age at the start of radiotherapy | | | | | | | | | | | |
| Linear (per 10 years) | 1.08 (0.96-1.22) | 1.08 (0.96-1.23) | - | - | 1.09 (0.96-1.23) | 1.08 (0.96-1.23) | 1.10 (0.96-1.26) | 1.17 (1.00-1.36) | 1.12 (0.98-1.28) | 1.09 (0.96-1.23) | 1.09 (0.96-1.23) |
| Categorical (50-69 relative to <50) | - | - | 1.21 (0.78-1.85) | - | - | - | - | - | - | - | - |
| Categorical (>=70 relative to <50) | - | - | 1.45 (0.91-2.32) | - | - | - | - | - | - | - | - |
| Squared | - | - | - | 1.00 (1.00-1.00) | - | - | - | - | - | - | - |
| Gross tumour volume (cm3) | | | | | | | | | | | |
| Log10 | 2.47 (1.73-3.53) | 3.05 (2.21-4.20) | 2.50 (1.75-3.57) | 2.47 (1.73-3.53) | - | 2.55 (1.77-3.65) | 2.62 (1.78-3.84) | 2.63 (1.69-4.08) | 2.92 (2.00-4.27) | - | 2.98 (2.17-4.09) |
| Categorical (50-99.99 relative to <49.99) | - | - | - | - | 1.79 (1.26-2.54) | - | - | - | - | - | - |
| Categorical (100-149.99 relative to <49.99) | - | - | - | - | 1.98 (1.21-3.24) | - | - | - | - | - | - |
| Categorical (>=150 relative to <49.99) | - | - | - | - | 3.20 (2.07-4.94) | - | - | - | - | - | - |
| Prescribed dose to primary tumour (per 10 Gy) | 1.17 (0.82-1.67) | 1.26 (0.88-1.81) | 1.18 (0.82-1.69) | 1.17 (0.82-1.67) | 1.17 (0.82-1.67) | 1.16 (0.81-1.67) | 1.14 (0.75-1.73) | 1.25 (0.77-2.05) | 1.06 (0.71-1.59) | 1.11 (0.78-1.57) | 1.23 (0.86-1.76) |
| Histology (Basaloid SCC relative to SCC) | 0.64 (0.39-1.06) | 0.63 (0.38-1.04) | 0.64 (0.39-1.06) | 0.64 (0.39-1.06) | 0.65 (0.39-1.07) | 0.64 (0.38-1.09) | 0.66 (0.38-1.12) | 0.65 (0.35-1.20) | 0.65 (0.38-1.11) | 0.64 (0.39-1.05) | 0.64 (0.39-1.05) |
| Chemotherapy regimen (all relative to no chemotherapy) | | | | | | | | | | | |
| Mitomycin-based chemotherapy | 0.67 (0.35-1.25) | 0.70 (0.37-1.31) | 0.67 (0.36-1.25) | 0.67 (0.36-1.26) | 0.71 (0.38-1.31) | 0.66 (0.35-1.24) | 0.75 (0.37-1.55) | 0.72 (0.36-1.47) | 0.71 (0.36-1.42) | 0.79 (0.42-1.48) | 0.69 (0.37-1.30) |
| Cisplatin-based chemotherapy | 0.72 (0.22-2.30) | 0.76 (0.24-2.43) | 0.74 (0.23-2.36) | 0.73 (0.23-2.33) | 0.78 (0.24-2.51) | 0.72 (0.22-2.30) | 0.72 (0.21-2.47) | 0.72 (0.21-2.4) | 0.74 (0.22-2.47) | 0.98 (0.31-3.12) | 0.74 (0.23-2.37) |
| Other chemotherapy regimens | 0.83 (0.30-2.27) | 0.91 (0.33-2.48) | 0.83 (0.30-2.26) | 0.83 (0.31-2.28) | 0.91 (0.33-2.49) | 0.74 (0.25-2.14) | 0.76 (0.25-2.31) | 0.44 (0.05-3.48) | 0.69 (0.22-2.19) | 0.93 (0.34-2.55) | 0.90 (0.33-2.44) |
| Radiotherapy technique (IMRT/VMAT relative to 3D-CRT) | 1.55 (0.91-2.64) | 1.61 (0.94-2.75) | 1.54 (0.90-2.63) | 1.54 (0.90-2.64) | 1.52 (0.89-2.60) | 1.64 (0.88-3.06) | 1.59 (0.78-3.25) | 1.67 (0.81-3.44) | 1.66 (0.81-3.40) | 1.40 (0.82-2.39) | 1.60 (0.93-2.73) |
| Performance status | | | | | | | | | | | |
| 1 relative to 0 | - | - | - | - | - | - | 1.42 (1.03-1.95) | - | - | - | - |
| 2 or 3 or 4 relative to 0 | - | - | - | - | - | - | 1.60 (0.92-2.77) | - | - | - | - |
| Completed treatment (Yes relative to No) | - | - | - | - | - | - | - | 0.79 (0.37-1.70) | - | - | - |
| Overall treatment time (days) | - | - | - | - | - | - | - | - | 1.02 (1.00-1.04) | - | - |

Supplementary Table 9. Summary of results from the secondary freedom from distant metastases models. CI: Confidence interval; GTV: Gross tumour volume; Gy: Gray; SCC: Squamous cell carcinoma.

| | Primary model | Secondary model 1 (Staging risk) | Secondary model 2 (Age categorical) | Secondary model 3 (Age squared) | Secondary model 4 (GTV categorical) | Secondary model 5 (GTV complete case) | Secondary model 6 (Performance status) | Secondary model 9 (No GTV) | Secondary model 10 (No T stage) |
|---|---|---|---|---|---|---|---|---|---|
| **Weighted mean c-index** | 0.69 | 0.68 | 0.69 | 0.69 | 0.68 | 0.69 | 0.69 | 0.67 | 0.68 |
| **Factor** | **Hazard ratio (95% CI)** | | | | | | | | |
| Nodal involvement (N+ relative to N0) | 2.09 (1.42-3.08) | - | 2.10 (1.43-3.10) | 2.09 (1.42-3.08) | 2.04 (1.38-3.02) | 2.14 (1.44-3.20) | 2.04 (1.33-3.13) | 2.24 (1.53-3.28) | 2.17 (1.48-3.17) |
| T stage (T3-4 relative to T1-2) | 1.18 (0.80-1.74) | - | 1.18 (0.80-1.74) | 1.18 (0.80-1.75) | 1.16 (0.78-1.72) | 1.32 (0.88-1.97) | 1.15 (0.74-1.78) | 1.65 (1.17-2.33) | - |
| Staging risk (High risk - [T4 Nany] or [Tany N+] relative to Low risk - [T1 or T2 or T3] and N0) | - | 2.10 (1.40-3.14) | - | - | - | - | - | - | - |
| Sex (Female relative to male) | 0.82 (0.58-1.16) | 0.80 (0.57-1.13) | 0.82 (0.58-1.16) | 0.82 (0.58-1.16) | 0.84 (0.60-1.19) | 0.83 (0.58-1.18) | 0.92 (0.62-1.38) | 0.82 (0.58-1.15) | 0.81 (0.58-1.15) |
| Age at the start of radiotherapy | | | | | | | | | |
| Linear (per 10 years) | 1.00 (0.86-1.16) | 1.00 (0.86-1.16) | - | - | 1.02 (0.88-1.18) | 1.00 (0.86-1.16) | 0.96 (0.81-1.13) | 1.01 (0.87-1.16) | 1.00 (0.86-1.16) |
| Categorical (50-69 relative to <50) | - | - | 1.20 (0.73-1.97) | - | - | - | - | - | - |
| Categorical (>=70 relative to <50) | - | - | 1.24 (0.71-2.15) | - | - | - | - | - | - |
| Squared | - | - | - | 1.00 (1.00-1.00) | - | - | - | - | - |
| Gross tumour volume (cm3) | | | | | | | | | |
| Log-transformed (log10) | 2.14 (1.40-3.27) | 2.32 (1.58-3.42) | 2.15 (1.41-3.30) | 2.14 (1.40-3.27) | - | 2.02 (1.32-3.11) | 2.19 (1.37-3.50) | - | 2.33 (1.59-3.40) |
| Categorical (50-99.99 relative to <49.99) | - | - | - | - | 1.54 (1.02-2.35) | - | - | - | - |
| Categorical (100-149.99 relative to <49.99) | - | - | - | - | 1.43 (0.74-2.74) | - | - | - | - |
| Categorical (>=150 relative to <49.99) | - | - | - | - | 3.37 (2.03-5.61) | - | - | - | - |
| Prescribed dose to primary tumour (per 10 Gy) | 1.21 (0.79-1.86) | 1.24 (0.80-1.90) | 1.22 (0.80-1.87) | 1.21 (0.79-1.86) | 1.23 (0.8-1.88) | 1.21 (0.78-1.86) | 1.41 (0.83-2.39) | 1.17 (0.77-1.78) | 1.23 (0.81-1.88) |
| Histology (Basaloid SCC relative to SCC) | 1.04 (0.64-1.69) | 1.03 (0.63-1.67) | 1.04 (0.64-1.69) | 1.04 (0.64-1.69) | 1.06 (0.65-1.73) | 1.21 (0.73-2.00) | 1.28 (0.77-2.13) | 1.03 (0.63-1.67) | 1.04 (0.64-1.69) |
| Chemotherapy regimen (all relative to no chemotherapy) | | | | | | | | | |
| Mitomycin-based chemotherapy | 0.59 (0.28-1.23) | 0.58 (0.28-1.23) | 0.60 (0.29-1.26) | 0.58 (0.28-1.23) | 0.65 (0.31-1.37) | 0.58 (0.27-1.21) | 0.56 (0.24-1.27) | 0.68 (0.33-1.42) | 0.59 (0.28-1.24) |
| Cisplatin-based chemotherapy | 0.80 (0.21-3.09) | 0.79 (0.21-3.07) | 0.83 (0.21-3.23) | 0.80 (0.21-3.09) | 0.90 (0.23-3.48) | 0.80 (0.21-3.08) | 0.76 (0.19-3.09) | 1.04 (0.27-3.97) | 0.81 (0.21-3.13) |
| Other chemotherapy regimens | 0.94 (0.31-2.92) | 0.89 (0.29-2.77) | 0.95 (0.31-2.94) | 0.94 (0.30-2.92) | 1.11 (0.36-3.45) | 0.78 (0.23-2.63) | 0.82 (0.24-2.85) | 1.03 (0.33-3.18) | 0.98 (0.32-3.01) |
| Performance status | | | | | | | | | |
| 1 relative to 0 | - | - | - | - | - | - | 1.55 (1.04-2.32) | - | - |
| 2 or 3 or 4 relative to 0 | - | - | - | - | - | - | 1.70 (0.83-3.51) | - | - |

## Supplementary Note 6

## Glossary

| Abbreviation | Definition |
|---|---|
| 3D-CRT | 3D Conformal Radiotherapy |
| 5FU | 5-Fluorouracil |
| Cap | Capecitabine |
| CDM | Common Data Model |
| CI | Confidence Interval |
| CORMAC | Core Outcome Research Measures in Anal Cancer |
| EQD2 | Equivalent Dose in 2 Gy Fractions |
| FFDM | Freedom from Distant Metastases |
| GPT | Generative Pre-trained Transformer |
| GTV | Gross Tumour Volume |
| IMRT | Intensity-Modulated Radiotherapy |
| LRC | Locoregional Control |
| MMC | Mitomycin C |
| OMOP | Observational Medical Outcomes Partnership |
| OS | Overall Survival |
| PLATO | PersonaLising Anal cancer radioTherapy dOse (trial) |
| REC | Research Ethics Committee |
| RWD | Real-World Data |
| SCC | Squamous Cell Carcinoma |
| TRIPOD | Transparent Reporting of a multivariable prediction model for Individual Prognosis Or Diagnosis |
| VMAT | Volumetric Modulated Arc Therapy |
| Vantage6 | Privacy-preserving federated learning infrastructure |
