# Supplementary material for: An international multi-centre study to develop and validate federated learning-based prognostic models for anal cancer
Source: Nat Commun. 2026 Mar 14;17:3956. doi: 10.1038/s41467-026-70297-3 (PMC13133161; doi:10.1038/s41467-026-70297-3)
Supplement: Supplementary file 3 — Reporting Summary [file 41467_2026_70297_MOESM3_ESM.pdf]

nature portfolio

Corresponding author(s): Stelios Theophanous

Last updated by author(s): 04/02/2025

Reporting Summary

Nature Portfolio wishes to improve the reproducibility of the work that we publish. This form provides structure for consistency and transparency in reporting. For further information on Nature Portfolio policies, see our Editorial Policies and the Editorial Policy Checklist.

Statistics

For all statistical analyses, confirm that the following items are present in the figure legend, table legend, main text, or Methods section.

n/a	Confirmed	
☐	☒	The exact sample size (*n*) for each experimental group/condition, given as a discrete number and unit of measurement
☐	☒	A statement on whether measurements were taken from distinct samples or whether the same sample was measured repeatedly
☒	☐	The statistical test(s) used AND whether they are one- or two-sided<br>*Only common tests should be described solely by name; describe more complex techniques in the Methods section.*
☐	☒	A description of all covariates tested
☐	☒	A description of any assumptions or corrections, such as tests of normality and adjustment for multiple comparisons
☐	☒	A full description of the statistical parameters including central tendency (e.g. means) or other basic estimates (e.g. regression coefficient) AND variation (e.g. standard deviation) or associated estimates of uncertainty (e.g. confidence intervals)
☒	☐	For null hypothesis testing, the test statistic (e.g. *F*, *t*, *r*) with confidence intervals, effect sizes, degrees of freedom and *P* value noted<br>*Give P values as exact values whenever suitable.*
☒	☐	For Bayesian analysis, information on the choice of priors and Markov chain Monte Carlo settings
☒	☐	For hierarchical and complex designs, identification of the appropriate level for tests and full reporting of outcomes
☒	☐	Estimates of effect sizes (e.g. Cohen's *d*, Pearson's *r*), indicating how they were calculated

Our web collection on statistics for biologists contains articles on many of the points above.

Software and code

Policy information about availability of computer code

Data collection	No software was used to collect data for this study.
Data analysis	The code developed for this study is publicly available at https://github.com/MaastrichtU-CDS/atomcat2 and is released under the GNU General Public License v3.0. The repository contains all scripts used for data processing, federated model execution, and aggregation of results. The code has been archived on Zenodo and assigned a persistent DOI: 10.5281/zenodo.18431456.<br><br>The open-source Vantage6 (v2.3.4) federated learning infrastructure was used to carry out all federated learning analyses: https://github.com/vantage6/vantage6. Two Vantage6 federated learning algorithms were employed to run the federated analysis in this study. Federated Cox regression algorithm: https://github.com/IKNL/vtg.coxph, and federated validation algorithm: https://github.com/MaastrichtU-CDS/vtg.coxph_val. Descriptive data analysis was conducted in each participating centre individually using R/RStudio, and aggregation of results (including federated results) was carried out by the coordinating centre using R/RStudio.<br><br>This information can be found in the "Federated model learning" section (page 12), in the "Code availability" section (page 13) , and in more detail in the Supplementary Material: Supplementary Note 3.

For manuscripts utilizing custom algorithms or software that are central to the research but not yet described in published literature, software must be made available to editors and reviewers. We strongly encourage code deposition in a community repository (e.g. GitHub). See the Nature Portfolio guidelines for submitting code & software for further information.

Data

Policy information about availability of data

All manuscripts must include a data availability statement. This statement should provide the following information, where applicable:

- Accession codes, unique identifiers, or web links for publicly available datasets
- A description of any restrictions on data availability
- For clinical datasets or third party data, please ensure that the statement adheres to our policy

Due to the nature of this research, the individual-level patient data analysed in this study cannot be made publicly available. These data include demographic variables, tumour characteristics, treatment details, and clinical outcomes collected at the participating centres. Even after de-identification, such data are considered potentially re-identifiable and fall outside the scope of the ethical approvals obtained for this study, which do not permit redistribution of patient-level data.

The study protocol and statistical analysis plan, including a detailed data dictionary defining all variables, have been previously published (Theophanous et al., Diagn Progn Res 2022; https://doi.org/10.1186/s41512-022-00128-8). Aggregated results supporting the findings of this study are provided in the figures and tables of the main manuscript and Supplementary Materials. Source data underlying the reported figures are provided with this paper.

This information can be found in the "Data availability" section (page 13) of the manuscript.

Research involving human participants, their data, or biological material

Policy information about studies with human participants or human data. See also policy information about sex, gender (identity/presentation), and sexual orientation and race, ethnicity and racism.

Reporting on sex and gender	The use of the terms "sex" and "gender" was discussed among the senior research team during the study design phase. A decision was made to use the term "sex" throughout the study, since biological and physiological characteristics of individuals were assessed.<br><br>Biological sex was included as a predictor in all multivariable models and was recorded based on clinical documentation at each participating centre. Gender identity was not separately collected or verified. Sex-specific analyses beyond inclusion as a covariate were not pre-specified and were not performed.<br><br>This information can be found in the Model Development & Specification subsection of the Methods section (page 11).
Reporting on race, ethnicity, or other socially relevant groupings	The study does not report on race, ethnicity, or other socially relevant groupings.
Population characteristics	Full characteristics of the patient population studied are reported in the "Results" section (pages 4-5) of the manuscript, and discussed further in the "Discussion" section (pages 6-8).
Recruitment	Patient data were identified and extracted from existing research and clinical databases at each participating centre. No patient recruitment took place for this study.<br><br>This information has been included in the "Patient data collection & missing data" section (page 11) of the manuscript.
Ethics oversight	The atomCAT2 study used FL methodology to develop prognostic models using local datasets from 16 participating centres (see Supplementary Material: Supplementary Note 2), without exchange of any sensitive individual patient-level data. Only non-identifiable aggregated information in the form of mathematical parameters, such as model coefficients, was shared between centres to train and validate the federated models. Each institution obtained approval from the relevant institutional review board or equivalent ethics committee for accessing and using patient data for research. Each participating centre provided a copy of their local data access approval, including approving body and reference number, to the central study coordinator. For UK centres, central approval was provided by the Health Research Authority (HRA) (IRAS project ID: 303103, REC reference: 22/WA/0081). Full details of the approving body, approval reference, and informed consent status for each centre are provided in Supplementary Note 2.<br>The atomCAT2 study involved no protocol-specific intervention beyond standard-of-care radiotherapy. Given the retrospective, non-interventional nature of the study and the use of routinely collected, de-identified data that remained at the originating institutions, the requirement for individual patient informed consent was waived or deemed not required by the approving bodies, as detailed in Supplementary Note 2.<br><br>This information can be found in the "Institutional data access & data protection approvals" section (page 9) of the manuscript and in Supplementary Note 2 (Supplementary Table 1).

Note that full information on the approval of the study protocol must also be provided in the manuscript.

Field-specific reporting

Please select the one below that is the best fit for your research. If you are not sure, read the appropriate sections before making your selection.

☒ Life sciences ☐ Behavioural & social sciences ☐ Ecological, evolutionary & environmental sciences

For a reference copy of the document with all sections, see nature.com/documents/nr-reporting-summary-flat.pdf

Life sciences study design

All studies must disclose on these points even when the disclosure is negative.

Sample size	A prospective sample size calculation was performed using the framework devised by Riley et al. (Stat Med 2019; 38: 1276–96), and implemented using the "pmsampsize" package in R, to determine the minimum sample size required to fit a Cox proportional hazards model for each of the three outcomes. The number of prognostic factors included in the final models was based on the total number of patients available in the primary consortium cohort; with the specific set of factors based on the prioritised list developed from a systematic literature review and expert oncologist input. The detailed methodology used to calculate sample size and prioritised list of factors is provided in the study protocol (https://diagnprognres.biomedcentral.com/articles/10.1186/s41512-022-00128-8).<br><br>Based on the final patient number available for model development and internal validation (n=1428), we included 11 parameters as predictors. An additional cohort of 277 patients was used for external validation.<br><br>This information can be found in the "Prospective study protocol and statistical analysis plan" section (page 11) of the manuscript.
Data exclusions	Exclusion criteria were pre-specified in the study protocol. Patients were excluded from the study cohort if they had undergone palliative treatment, if they had received prior pelvic radiotherapy, or if they had received brachytherapy (either primary or as boost treatment). In addition, the study protocol pre-specified a framework on how to deal with missing data at individual centres, in multiple different scenarios, including use of data imputation.<br><br>This information is included in the "Study design & patient population" section (page 10), as well as the "Patient data collection & missing data" section (page 11) of the manuscript.
Replication	Federated learning models were initially validated through internal 'leave-one-centre-out' validation, where models were optimised on data from all but one centre and evaluated on the out-of-sample centre data. This was repeated for all potential centre combinations, and average out-of-sample model performance was reported. Subsequently, model performance was evaluated on an external, unseen dataset from two additional centres not involved in the primary analysis.<br><br>This information can be found in the "Evaluation and visualisation of model performance" (page 12) and "External validation" (pages 12-13) sections of the manuscript.
Randomization	No allocation of patients into experimental groups took place. Covariates were controlled by developing multivariable models. The most relevant prognostic factors were prioritised through a systematic review of the literature. Factors identified as prognostic through multivariable analysis in several studies were selected and prioritised (based on the number of studies reporting on them), which formed an initial list of relevant data to be collected. This list was reviewed by three senior clinical oncologists (AG, MGG, MB), who added additional relevant factors, and created a prioritised list of factors for each prognostic model.<br><br>This information is included in the "Identification of relevant prognostic factors" section (page 10) of the manuscript.
Blinding	Patients were not allocated to groups, and therefore blinding was not relevant to this study.

Reporting for specific materials, systems and methods

We require information from authors about some types of materials, experimental systems and methods used in many studies. Here, indicate whether each material, system or method listed is relevant to your study. If you are not sure if a list item applies to your research, read the appropriate section before selecting a response.

Materials & experimental systems

n/a	Involved in the study	
☒	☐	Antibodies
☒	☐	Eukaryotic cell lines
☒	☐	Palaeontology and archaeology
☒	☐	Animals and other organisms
☐	☒	Clinical data
☒	☐	Dual use research of concern
☒	☐	Plants

Methods

n/a	Involved in the study	
☒	☐	ChIP-seq
☒	☐	Flow cytometry
☒	☐	MRI-based neuroimaging

Clinical data

Policy information about clinical studies

All manuscripts should comply with the ICMJE guidelines for publication of clinical research and a completed CONSORT checklist must be included with all submissions.

Clinical trial registration	N/A - not a clinical trial. Study was prospectively registered with the Open Science Foundation (https://osf.io/xhbv8/)
Study protocol	Published open access in the Diagnostic and Prognostic Research journal: https://diagnprognres.biomedcentral.com/articles/10.1186/s41512-022-00128-8<br><br>Each institution obtained approval from the relevant institutional review board or equivalent ethics committee for accessing and using patient data for research. Each participating centre provided a copy of their local data access approval, including approving body and reference number, to the central study coordinator. For UK centres, central approval was provided by the Health Research Authority (HRA) (IRAS project ID: 303103, REC reference: 22/WA/0081). Full details of the approving body, approval reference, and informed consent status for each centre are provided in Supplementary Note 2.<br>The atomCAT2 study involved no protocol-specific intervention beyond standard-of-care radiotherapy. Given the retrospective, non-interventional nature of the study and the use of routinely collected, de-identified data that remained at the originating institutions, the requirement for individual patient informed consent was waived or deemed not required by the approving bodies, as detailed in Supplementary Note 2.<br><br>This information can be found in the "Institutional data access & data protection approvals" section (page 9) of the manuscript and in Supplementary Note 2 (Supplementary Table 1).
Data collection	Patient data from January 2004 to December 2024 were identified and extracted from existing research and clinical databases at each participating site. To ensure good data quality, each institution spot checked all extracted data, ensuring adherence to the coding system specified in the data dictionary and identifying any outliers. Central review of summary patient, treatment and outcome data from each centre also provided an additional level of quality assurance.<br><br>This information is included in the "Patient data collection & missing data" section (page 11), as well as the "Results" section (pages 4-5) of the manuscript.
Outcomes	Three outcomes were explored: overall survival, locoregional control and freedom from distant metastasis. These were identified as key outcome research measures in anal cancer by the CORMAC initiative (Lancet Gastroenterol Hepatol. 2018 Dec;3(12):865-873 and EClinicalMedicine. 2024 Dec 5;78:102939).<br><br>Overall survival<br>Overall survival was calculated in days from the first fraction of radiotherapy to either event or censoring, whichever happened first. An event was defined as death from any cause at any point during follow-up. Patients were censored at the last clinical follow-up date if alive.<br><br>Locoregional control<br>Time to locoregional control was calculated in days from the first fraction of radiotherapy to either event or censoring, whichever happened first. An event was defined as any of the following as a first event: (1) Abdominoperineal resection to control locoregional disease at any point during follow-up. This always took precedence in terms of date for locoregional recurrence. (2) Locoregional disease progression, during treatment or in follow-up (irrespective of whether complete or partial response have been initially achieved), not managed by surgery. This was preferably confirmed with biopsy, in which case the date of biopsy was used but was alternatively based on imaging and clinical examination only (date of imaging was used). (3) Lack of complete response (non-clearance of disease) at 26 weeks (6 months) from first fraction of radiotherapy, as defined by clinical examination, imaging and/or biopsy. In case of uncertainty or where limited information was available, the date where treatment failure or locoregional recurrence was first noted in the patient records was used.<br>Patients were censored at death, at last clinical follow-up, if undergoing abdominoperineal resection for non-disease related reasons (e.g. due to treatment complications), or in case of distant metastases.<br><br>Freedom from distant metastasis<br>Freedom from distant metastasis was calculated in days from the start of radiotherapy to either event or censoring, whichever happened first. An event was defined as distant disease recurrence (previously untreated lymph node metastasis outside the pelvis, or other metastatic sites such as lung, liver, bone) as a first event. This may have been confirmed with biopsy, in which case the date of biopsy was used as the date of recurrence, or alternatively based on imaging (date of imaging was used). In case of any uncertainty or where limited information was available, the date where distant progression was first noted in the patient records was used. Site(s) of failure were noted. Patients were censored at local recurrence, at death, or at last clinical follow-up.<br><br>This information can be found in the "Outcome definitions" section (page 10) of the manuscript, which also directs the reader to the relevant section in the study protocol.

Plants

Seed stocks	N/A
Novel plant genotypes	N/A
Authentication	N/A
